# Pandemic *Vibrio parahaemolyticus* O3:K6 Spread, France

**DOI:** 10.3201/eid1107.041008

**Published:** 2005-07

**Authors:** Marie-Laure Quilici, Annick Robert-Pillot, Jessica Picart, Jean-Michel Fournier

**Affiliations:** * Institut Pasteur, Paris, France

**Keywords:** France, molecular typing, O3:K6 serovar, pandemic clone, Vibrio parahaemolyticus

**To the Editor:** *Vibrio parahaemolyticus* is a halophilic bacterium that occurs naturally in aquatic environments worldwide. It causes one of the most severe forms of gastroenteritis and is the leading cause of seafood-associated bacterial gastroenteritis in the world, often associated with the consumption of raw or undercooked seafood. Since 1996, the incidence of *V*. *parahaemolyticus* infections has increased dramatically. *V*. *parahaemolyticus* strains previously associated with only sporadic cases of gastroenteritis have caused large-scale outbreaks in North America and epidemics in India, Southeast Asia, and Japan (1). This increase in incidence appears to be related to the emergence of a new clone, belonging to the O3:K6 serovar, which has pandemic potential. This clone was named the new O3:K6 clone to distinguish it from strains belonging to this serovar isolated before 1996, which are less pathogenic. We report the first evidence for the presence in France and suggest the presence and persistence in French coastal areas of this pandemic O3:K6 serovar, which is indistinguishable from the O3:K6 clone isolated in Bangladesh in 1996.

We analyzed 13 clinical isolates of *V*. *parahaemolyticus* collected in France from 1997 to 2004 and sent to the National Reference Center (Table). All isolates were characterized by polymerase chain reaction (PCR) to detect the genes encoding the virulence-associated hemolysins, thermostable-direct hemolysin, and thermostatable-related hemolysin (2), and 2 other genetic markers, *toxRS* and *orf8* (1,3). We also carried out molecular typing by various methods, including ribotyping, pulsed-field gel electrophoresis (PFGE), and arbitrarily primed PCR. Strains were initially identified by biochemical and cultural methods. Strain identities were confirmed by species-specific R72H PCR (4). The slide agglutination test was performed to determine whether the isolates belonged to the O3:K6 serovar. Two pandemic strains of the new O3:K6 clone and 1 strain of the old O3:K6 clone were included as external controls.

**Table:** Characteristics of the Vibrio parahaemolyticus O3:K6 strains studied*

Strain no. CNRVC (source no.)	Origin	Date of isolation	Source of transmission	Detection of gene or phage sequences by PCR							
				*R72H*	*tdh*	*trh*	*toxRS*	*orf8*	Ribotype profile	PFGE profile	AP-PCR profile
970136	France (Atlantic coast)	Oct 1997	Local oysters	+	+	-	+	+	R4†	P-1a	AP-a
980402	France (southwest)	Sep 1998	Shellfish	+	+	-	+	+	R4	P-1c	AP-a
990346	France (Mediterranean coast)	Aug 1999	–	+	+	-	+	+	R4	P-1c	AP-a
030478	France (Atlantic coast)	Aug 2003	Local shellfish	+	+	-	+	+	R4	UT‡	AP-a
030479	France (Atlantic coast)	Aug 2003	–	+	+	-	+	+	R4	UT	AP-a
020468 (AN7410)	Bangladesh	1998		+	+	-	+	+	R4	P-1a	AP-a
020469 (AO1851)	Bangladesh	1999		+	+	-	+	+	R4	P-1b	AP-a
030085 (AQ4037)	Maldives	1985		+	-	+	-	-	Rb-2§	P-2	AP-b

*CNRVC, Centre National de Référence des Vibrions et du Cholera; PCR, polymerase chain reaction; PFGE, pulsed-field gel electrophoresis; AP-PCR, arbitrarily primed PCR. †R4 ribotype pattern as described previously (5). ‡UT, untypeable: DNA was degraded before PFGE, presumably by DNases. §According to our pattern designation.

Five strains were identified as *V*. *parahaemolyticus* O3:K6 by slide agglutination test. With the exception of the strain referred to as old O3:K6 clone, all O3:K6 strains studied, whether isolated in France or included as controls, were positive for *tdh*, *toxRS,* and *orf*8 genes. Likewise, all strains, except the old O3:K6 clone, produced *Bgl*I rRNA gene restriction patterns identical to those of a major R4 ribotype previously described (5) and were genetically indistinguishable by arbitrarily primed PCR analysis. With the exception of the strain belonging to the old O3:K6 clone, the PFGE-typeable O3:K6 strains, despite having slightly different *Not*I patterns that reflect genetic rearrangement, clearly belonged to a single clone.

Until recently, *V*. *parahaemolyticus* caused only sporadic diarrhea and was never associated with a pandemic. The epidemiology of this organism changed abruptly after the new O3:K6 strains appeared in 1996. The spread of this serotype signaled the beginning of the first *V*. *parahaemolyticus* pandemic. Because the pathogenic *V*. *parahaemolyticus* O3:K6 isolates in France are derived from the new O3:K6 clone initially described in Bangladesh, this population likely was transported here in the same manner as *V*. *parahaemolyticus* O3:K6 was introduced into US coastal waters (6) and *V*. *cholerae* serogroup O1 was introduced into Gulf Coast waters in 1991 (7).

Epidemiologic information was collected from all patients with a standardized questionnaire concerning clinical history, symptoms, and seafood consumption. Responses indicated that some persons affected by *V*. *parahaemolyticus* O3:K6 had eaten local seafood harvested in uncontrolled areas. Furthermore, some had eaten seafood harvested in the same place several years apart. This provides evidence that pathogenic *V*. *parahaemolyticus* is present and suggests that it can persist in the French coastal environment.

The consumption of raw and lightly cooked seafood is increasing, as is the number of susceptible persons, which causes concern that the incidence of *V*. *parahaemolyticus* infections in Europe will increase. Monitoring this foodborne illness is difficult because only cases involving severe gastroenteritis are reported. Estimates are that only 1 in 20 cases of bloody diarrhea and only 1 in 38 cases of nonbloody diarrhea are reported in the United States (8). In France, the official surveillance authority estimated that the number of cases reported by the National Reference Center was representative of severe *Vibrio* infections. Making *Vibrio* isolations and infections reportable could help us estimate the true incidence of the disease and could improve the surveillance of *V*. *parahaemolyticus* infections.

Detecting the pathogenic *V*. *parahaemolyticus* O3:K6 in France, and previous results showing that pathogenic *V*. *parahaemolyticus* strains are present in French coastal areas at a higher frequency than was usually reported (9), may provide an early warning. Much effort is required to develop *V*. *parahaemolyticus* prevention strategies. Educating consumers about basic principles of food safety, particularly storage conditions, is an important component of prevention. Lack of continuous refrigeration from harvest to consumption may have contributed to these infections. The number of bacteria in seafood contaminated with only a small number of *V*. *parahaemolyticus* organisms can reach the infectious dose, thought to be >10^5^ CFU per gram according to the Centers for Disease Control and Prevention (10), within a few hours when left in a warm place. Another component of prevention is the improvement of microbial surveillance by systematic testing for pathogenic *V*. *parahaemolyticus* isolates in the environment and in locally produced and imported seafood.
